# Communication delay increases procedure time and instructor task load during simulated suturing by novices

**DOI:** 10.1038/s41526-026-00610-3

**Published:** 2026-06-13

**Authors:** Albert Yeam, Khushi Bhatt, Katherine Hanna, Tovy Haber Kamine, Ariana M. Nelson

**Affiliations:** 1 School of Medicine, University of California, Irvine, CA USA; 2 Division of Trauma, Acute Care Surgery, and Surgical Critical Care, Baystate Medical Center, Springfield, MA USA; 3 Department of Anesthesiology & Perioperative Care, University of California, Irvine, Orange, CA USA

**Keywords:** Health care, Medical research

## Abstract

Communication delay during remote instruction of simple interrupted suturing was evaluated by randomizing participants to near real-time or 4-second delayed communication conditions. The delay group required more time to complete five sutures, with diminishing effect by the fifth suture. Instructor-reported cognitive workload was higher under delayed conditions, but suturing quality did not differ between groups. These findings suggest remote guidance is operationally feasible for non-medically trained individuals performing uncomplicated interventions.

As humanity strives to become interplanetary, delays in communication remain a significant challenge. While astronauts receive basic training in first-line medical procedures, the National Aeronautics and Space Administration’s (NASA) current operational paradigm relies heavily on real-time mission control healthcare staff for more complex tasks or in emergencies^1^. However, as exploration class missions move beyond low Earth orbit to the Moon and Mars, increased distance from Earth results in higher latency communications, limited access to ground resources, and fewer opportunities for evacuation. For the Artemis missions, one-way lunar communications are projected to have a latency of 3 to 14 s. This is due to the impact of not only distance but also delays in the Deep Space Network, the telecommunication system that permits communications with extraterrestrial sites^2,3^. Despite this, there are limited studies evaluating the impact of one-way delays >1 s simulating lunar missions, leading to significant uncertainty in the projected risks^2^.

For routine evaluation and management of medical issues, a 3 to 14 s delay may be inconsequential, but for acute procedural oversight, these delays can have a considerable impact. Prior studies have shown that moderate delays (>700 ms) during telementored procedures degrade operator performance and increase task burden of both mentor and operator during simulated surgical scenarios^4^. However, none to date has shown the impact of a cis-lunar communication delay on an acute care scenario likely to occur during a month-long mission, such as an injury requiring suture for wound closure.

Understanding the ramifications of communication delays on inexperienced operators is a first step in developing solutions. To assess the impact of an intermediate communication delay on remotely instructing untrained operators, we simulated a suturing task with pre-medical students with and without a delay of 4 s.

Nine students were recruited. One participant was excluded due to having a functional movement disorder, and the remaining students were randomized to near real-time (NRT) (*n* = 4) or delayed groups (*n* = 4). All participants were right-handed, had never received prior instruction in suturing or surgical knot-tying, and had no prior experience learning with a communication delay. The average age did not differ significantly between groups (NRT: 21.75 ± 3.0 years vs. delayed: 22.5 ± 2.1 years, *P* = 0.69). Differences in pre-survey responses assessing participant confidence, ability, and interest were not statistically significant (Table 1).

**Table 1:** Comparison between pre-survey results between near real-time and delayed groups

	Near real-time group median (IQR)	Delayed group median (IQR)	U statistic	*P* value
I feel confident in my fine motor skills.	4 (4–4)	3.5 (3–4)	4	0.429
I feel confident in my ability to learn hands-on technical skills.	4 (4–4.75)	4 (4–4)	6	>0.999
I feel confident in my ability to perform a basic interrupted suture.	3.5 (3–4)	3 (2.25–3.75)	5	0.657
I feel comfortable using surgical instruments (e.g., forceps, needle driver).	3 (2.25–3.75)	2.5 (1.25–3)	4.5	0.486
I feel nervous about performing a hands-on medical procedure.	3.5 (3–4)	4 (3.25–4)	6	>0.999
I am interested in pursuing a surgical or procedural medical specialty.	3.5 (2.25–4.75)	3.5 (2.25–4.75)	8	>0.999

Pre- and Post-surveys used a Likert Scale (1 – strongly disagree, 2 – disagree, 3 – neutral, 4 – agree, 5 – strongly agree).

A delay significantly increased the time to complete sutures (Fig. 1). The first suture required an average of 338.4 ± 77.6 s in the NRT group versus 633.5 ± 198.9 s in the delayed group (*P* = 0.03). A similar pattern was observed for the second suture (139.1 ± 48.2 s vs. 316.9 ± 59.0 s, *P* < 0.01) and fourth suture (115.9 ± 26.0 s vs. 187.8 ± 50.4 s, *P* = 0.04). The cumulative time to complete all five sutures was nearly doubled in the delay group (791.3 ± 168.6 s vs. 1471 ± 360.8 s, *P* = 0.01). There were no statistically significant differences for the 3rd and 5th sutures individually.

**Fig. 1:**
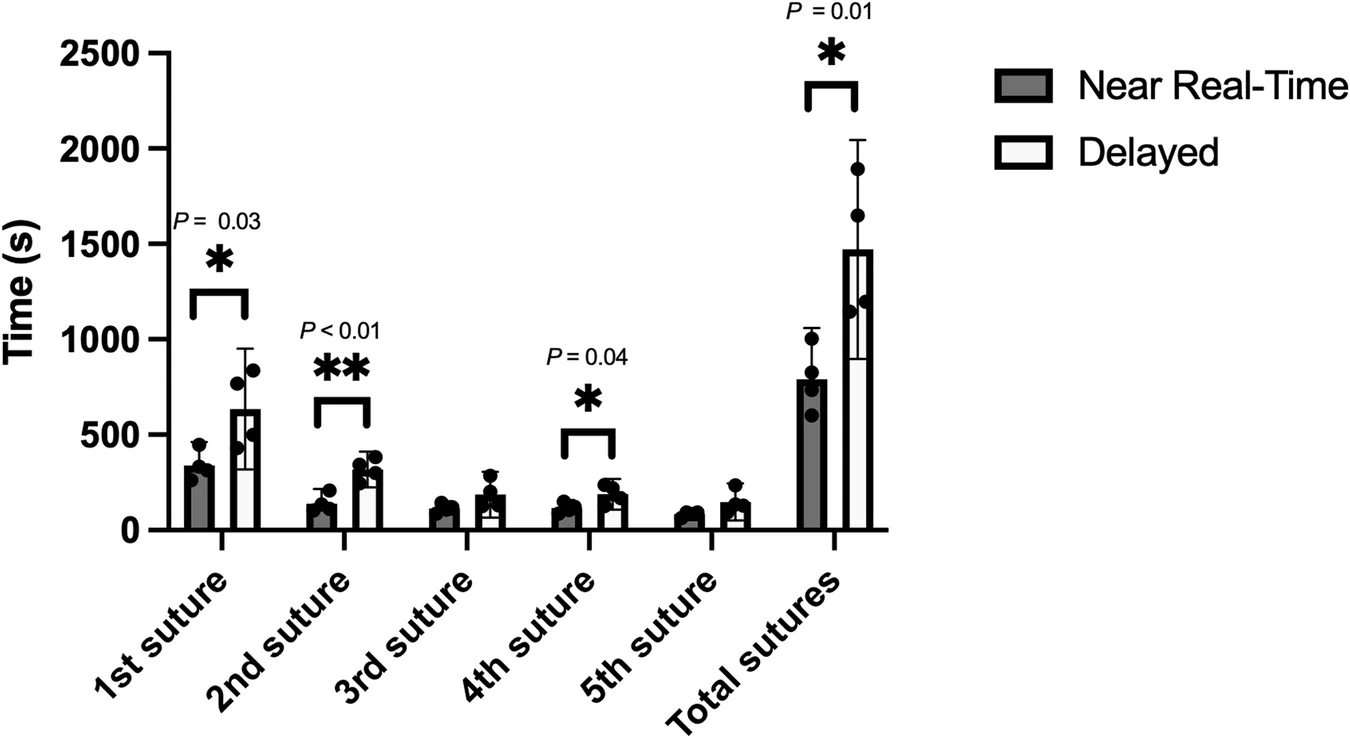
Comparison of suturing times between near real-time and delayed groups. The cumulative and individual suturing times were compared between near real-time and delayed groups using unpaired student’s *t*-tests. Error bars represent 95% confidence intervals.

Performance, as measured by blinded Observed Structured Assessment of Technical Skill (OSATS) scores, was not significantly lower in the delayed group. The average OSATS score (out of 8) was 6.0 ± 1.4 in the NRT group vs. 4.63 ± 0.6 in the delayed group (*P* = 0.13).

NASA Task Load Index (TLX) scores were used as a subjective assessment of workload on participants and the instructor. For participants in both groups, the average and individual TLX scores were not significantly different. However, for the instructor, statistical analysis revealed significant differences between NRT and delayed groups in mental (58.8 ± 11.1 vs. 86.3 ± 6.3, *P* < 0.01), effort (63.7 ± 6.3 vs. 85 ± 10.8, *P* = 0.01), and frustration (43.8 ± 8.5 vs. 85 ± 9.1, *P* < 0.001) dimensions and overall TLX scores (46.8 ± 8.4 vs. 65 ± 3.6, *P* < 0.01) (Fig. 2). Additionally, TLX scores were compared between participants and the instructor. In the delayed group, the instructor experienced greater mental workload (86.3 ± 6.3 vs. 70 ± 4.1, *P* < 0.01) and frustration (85 ± 9.1 vs. 41.3, *P* < 0.05) compared to participants. Participants experienced a greater physical workload compared to the instructor (28.8 ± 12.5 vs 8.8 ± 2.5, *P* < 0.05). The overall workload did not differ between participants and the instructor in the delayed group (50 ± 14.0 vs. 65 ± 3.6, *P* = 0.08).

**Fig. 2:**
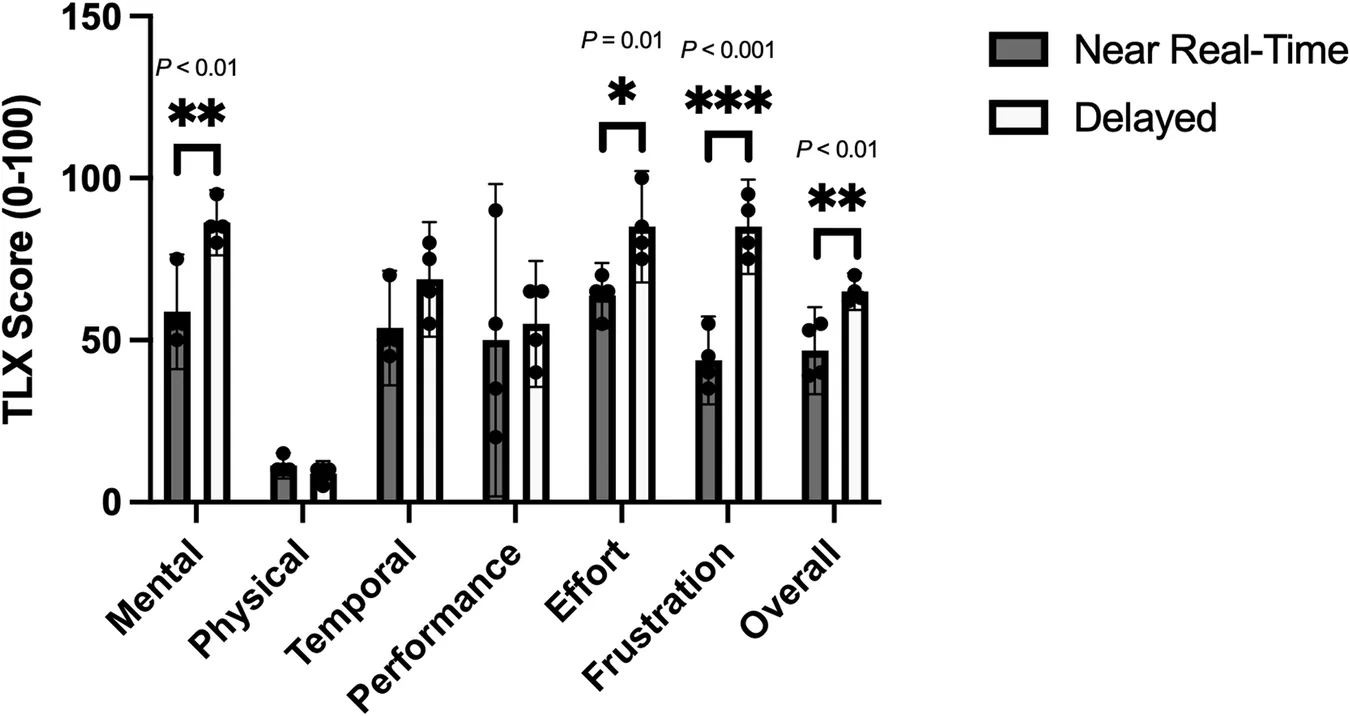
Comparison of instructor NASA task load index between near real-time and delayed groups. The NASA Task Load Index questionnaire was used to assess workload in six dimensions. The overall score was the average of all six dimensions. Scores were compared with unpaired student’s *t*-tests. Error bars represent 95% confidence intervals.

In the post-survey, there were no significant differences in the participants’ self-reported confidence, ability, or learning (Table 2). However, all participants in the delayed group agreed that the 4-s latency affected their ability to learn the procedure (3 agreed, 1 strongly agreed).

**Table 2:** Comparison between post-survey results between near real-time and delayed groups

	Near real-time group median (IQR)	Delayed group median (IQR)	U statistic	*P* value
I feel confident in my ability to perform a basic interrupted suture.	4 (3.25–4)	4 (3.25–4.75)	6.5	>0.999
I feel comfortable using surgical instruments (e.g., forceps, needle driver).	3.5 (2.25–4)	3 (3–3.75)	7.5	>0.999
My suturing technique improved during the session.	4.5 (4–5)	5 (2.75–5)	7	>0.999
I would feel comfortable practicing suturing independently after this session.	3.5 (2.25–4)	4.5 (2.5–5)	4.5	>0.999
I feel confident in my ability to learn hands-on technical skills.	4.5 (4–5)	4.5 (4–5)	8	>0.999
The instruction or guidance was clear and easy to follow.	4.5 (3.25–5)	3.5 (3–4.75)	5.5	>0.999
The pace of the session was appropriate.	4.5 (4–5)	4.5 (3.25–5)	7	>0.999
I felt frustrated during the session.	2 (1.25–2.75)	3 (1.5–3.75)	4.5	>0.999
Receiving remote instruction affected my ability to learn (compared to in-person).	4 (2.5–4)	4 (2.5–4.75)	6.5	>0.999
How would you rate your suturing technique at the end of the session?	3 (2.25–3.75)	3 (2.25–3.74)	8	>0.999

Pre- and Post-surveys used a Likert Scale (1 – strongly disagree, 2 – disagree, 3 – neutral, 4 – agree, 5 – strongly agree).

One of the most likely extraterrestrial scenarios requiring immediate procedural intervention is an injury requiring suture. Determining the impact of communication delays on suture completion and task load is critical for risk mitigation in space exploration^5^. Our study demonstrates that remotely instructing simple interrupted sutures with a 4-s communication delay decreases the overall operator efficiency. These results are consistent with previous studies demonstrating that communication delays impair telementored procedural performance, with effects dependent on delay duration. Earlier studies using subsecond delays demonstrated that communication delay strains were primarily borne by the mentor, with significant increases in mental demand and effort but little change in operator workload^6^. In our study, this pattern appeared in the delayed group, where the instructor reported higher mental load and frustration, while participants reported higher physical workload compared to the instructor, consistent with the instructor’s limited physical engagement. Unlike object-removal tasks that require constant feedback, suturing is repetitive, allowing greater within-session learning and reduced reliance on the mentor, which may explain why OSATS scores did not differ significantly between groups. Although a significant difference was noted at the fourth suture, both groups demonstrated progressively faster completion times with successive sutures, as well as a decreasing gap between groups, consistent with a learning effect and increasing independence from the instructor.

Other telementoring studies provide further context. A recent study in simulated appendectomy training showed that initial performance deficits with virtual instruction diminished over time, suggesting that structured preparation may mitigate early impairment^4^. However, only emergency medicine physicians were recruited for this study, restricting generalizability to similar provider populations. Although direct comparisons are constrained by differences in participant experience and task complexity, our findings suggest that basic procedural skill acquisition remains feasible for novices despite substantial communication latency. Comparisons across different provider experience levels indicate that skill level strongly influences procedural efficiency and resource cost under delayed communication^7^. In our inexperienced cohort, OSATS scores between our standard and delayed groups were similar, but suboptimal, even for a simple procedure. This is not surprising given previous studies showing that the number of sutures required to develop competency is 74^8^. Additional research is needed to explore how varying expertise interacts with latency to shape learning curves and technical performance.

While our study focused on first-time learners, prior research has examined the impact of previous learning on procedural skill acquisition and retention. In a simulation study of medical students who initially learned cardiopulmonary resuscitation, mean skill progressively declined: 90% at 1 month, 74% at 18 months, 62% at 30 months, and 61% at 42 months (*P* < 0.001), demonstrating skill decay without continued practice^9^. Conversely, medical students with prior intravenous cannulation training completed the procedure significantly faster during relearning 6 months later compared to those without prior training (108 s vs. 168 s; *P* = 0.006)^10^. In another trial, medical students taught chest tube placement via an instructional video who were subsequently randomized to face-to-face training or remote training showed superior procedural performance compared with those without additional instruction, despite similar procedural knowledge between groups^11^. In an extraterrestrial situation, procedural performance may be enhanced through prior learning reinforced by remote instruction, analogous to just-in-time simulation training, which rehearses a task at the time of need and has been shown to improve clinical preparedness^12^. Follow-up studies could examine the impact of remote instruction on long-term skill maintenance. This framework may inform the development of medical training for astronauts and remote health care providers and supports the inclusion of basic procedural training in crew medical officer training.

Future research could extend to other interventions (i.e., ultrasound, simulated intubation or lumbar puncture) to determine whether the results are consistent. The NASA Integrated Medical Model predicts the most likely medical outcomes to occur in space flight, and assessing how time delays impact treatment of these conditions is essential^13^. Limitations of our study include a small sample size, a lack of longitudinal assessment of retention, and a single-camera vantage point, which can be addressed in future studies.

In conclusion, we found that for inexperienced operators, a 4-s communication delay increases the overall time and instructor workload in a simple interrupted suture, but did not significantly change performance. Our results build on the existing literature evaluating the effect of intermediate communication delays on remote instruction and teleprocedures. It may be relatively safe for crew members to complete minimally invasive procedures, such as suturing small wounds, with intermediately delayed telemonitoring, even without prior simulation training.

## Methods

This study was approved by the University of California, Irvine Institutional Review Board with an exemption and waiver of informed consent (IRB #2025-7284) and was performed in accordance with the Declaration of Helsinki. We conducted a single-center, randomized controlled trial in which undergraduate-level students without professional medical training or suturing experience were telementored during the performance of simple interrupted sutures. One group was telementored NRT (400 ms) and the other with an audio and visual delay (4 s) over a video platform (Zoom © version 6.5.1). The 400 ms delay in the NRT group was due to the inherent latency in using video communication platforms and was determined by using a web-based world clock with accuracy to ms. Screenshots were taken on the instructor’s and participants’ computers to calculate the delay.

Eligible subjects were at least 18 years of age, pre-medical undergraduate students and had never previously received instruction on suturing or surgical knot-tying. Students were recruited via email that explained the study's purpose, potential risks involved, and the voluntary nature of participation. Participants were informed that they would learn a medical procedure, but the procedure type was not disclosed until initiation of the study procedures to prevent preparatory learning. Subjects were block randomized into groups of four to be equally distributed into the two study groups.

Participants and the telementor were blinded to whether they would be learning NRT or with delay. Participants provided written informed consent prior to completing a pre-procedure survey assessing their baseline confidence and perceived ability relating to suturing skills.

Study material included a suturing pad, needle driver, forceps, surgical scissors, and a 4-0 nylon monofilament with a 3/8 circle reverse cutting needle. A laptop with a videoconferencing application (Zoom © version 6.5.1) was mirrored onto a larger television screen, from which a remote telementor observed and provided direct psychomotor instruction and feedback on the participant’s suturing skills. The OBS Studio application (Open Broadcaster Software version: 31.1.2) applied a 4-s audio and visual delay over Zoom. Participants were instructed to perform five simple interrupted sutures in a 30-min time frame under the guidance of a general surgeon with extensive experience in clinical education. The time to complete each suture was recorded. Each performance was video recorded, and two independent blinded raters assessed performance using a standardized checklist derived from a suturing OSATS (Supplemental Table 1)^14^. The two independent raters were experienced clinical educators, consisting of a general surgeon and an anesthesiologist. Post-study, each participant completed a post-procedure survey reassessing their perceived performance. Additionally, both the instructor and participant completed the NASA TLX survey, a subjective workload assessment that has been validated in a healthcare setting^15,16^.

Outcomes assessed in this study included the time to complete each individual suture, the overall time to complete all five sutures, instructor and participant NASA TLX scores, and participant pre- and post-survey responses. The NASA TLX consists of six dimensions, including mental demand, physical demand, temporal demand, performance, effort, and frustration. Each dimension has a workload scale of 0–100. The overall workload was the average of all six dimensions. The participant pre- and post-surveys used a 5-point Likert scale (1 = strongly disagree, 2 = disagree, 3 = neutral, 4 = agree, 5 = strongly agree).

Given limited prior data and uncertainty regarding the expected effect size, a formal power analysis was not conducted. All eligible participants during the study period were included. Statistical analyses were conducted via the application GraphPad Prism version 10.5.0. Participants’ age, times to complete each suture, total suturing time, OSATS scores, and each dimension of the NASA TLX were averaged and compared between groups using unpaired student’s *t*-tests. Additionally, NASA TLX dimensions were compared between participants and instructor for NRT and delayed groups. Unpaired two-tailed *t*-tests were used to compare group means because observations were independent, measurements were continuous variables, and both groups demonstrated approximate normality (Shapiro–Wilk, *P* > 0.05). Data is presented as mean ± SD. The differences between pre- and post-survey data between groups were evaluated using a Mann–Whitney test, due to the data being ordinal.

## Supplementary information

Supplementary Information

## Data Availability

All data generated or analyzed during this study are included within the manuscript.
