# Supplementary material for: Communication delay increases procedure time and instructor task load during simulated suturing by novices
Source: NPJ Microgravity. 2026 Jun 13;12:83. doi: 10.1038/s41526-026-00610-3 (PMC13623917; doi:10.1038/s41526-026-00610-3)
Supplement: Supplementary file 1 [file 41526_2026_610_MOESM1_ESM.pdf]

Supplemental Table 1. Modified OSATS Checklist

ITEM	Not done correctly	Done correctly
**SUTURING – Basic technique**		
1. Holds needle driver properly (thumb and long/ring finger with index as stabilizer)	0	1
2. Loads needle properly (at tips of jaws, 1/2 to 2/3 from point)	0	1
3. Needle enters perpendicular to skin	0	1
4. Bites are symmetrical	0	1
5. Passes needle through tissue without sawing, following curve of needle	0	1
**SUTURING – Simple interrupted skin closure**		
1. No gap between wound edges	0	1
2. Everts wound edges	0	1
3. No dog ear at ends	0	1
